# Combined Laparoscopic Open Surgical Approach for De Garengeot’s Hernia Containing an Inflamed Appendix: A Case Report

**DOI:** 10.7759/cureus.46877

**Published:** 2023-10-11

**Authors:** Mostafa Elkhawaga, Ahmad S Alam, Amro Eldomiaty

**Affiliations:** 1 Surgery, John Hunter Hospital, Newcastle, AUS; 2 General Surgery, John Hunter Hospital, Newcastle, AUS; 3 Emergency Department, Al-Rwad Specialized Hospital, Menouf, EGY

**Keywords:** rare form of femoral hernia, femoral hernia, acute appendicitis, de garengeot's hernia, incarcerated femoral hernia

## Abstract

De Garengeot hernia represents a rare variant of femoral hernia in which the appendix is a part of the hernial contents. It was first described in 1731 by a French surgeon, René de Garengeot. In 1785, Hevin was the first to perform an appendectomy to address acute appendicitis within the context of a femoral hernia. The development of acute appendicitis in the femoral hernia sac becomes a surgical emergency of the acute abdomen.

## Introduction

A de Garengeot hernia is usually an unexpected finding during surgical procedures. It is observed in a range of 0.5% to 3% of all cases of femoral hernias [1]. The occurrence of acute appendicitis within femoral hernia is estimated based on statistics to range between 0.13% and 1% of all cases of acute appendicitis [2]. De Garengeot hernia is rarer than Amyand hernia (inguinal hernia containing an appendix) [3]. The presence of a pelvic appendix, or an enlarged, highly mobile cecum extending into the pelvis, may facilitate the entrapment of the appendix within a femoral hernia, causing De Garengeot hernia [4]. The preoperative diagnosis poses significant challenges due to the infrequency of these cases and the absence of typical symptoms [5]. This paper reports a rare case of incarcerated femoral hernia containing the appendix inside his sac.

## Case presentation

**Figure 1:**
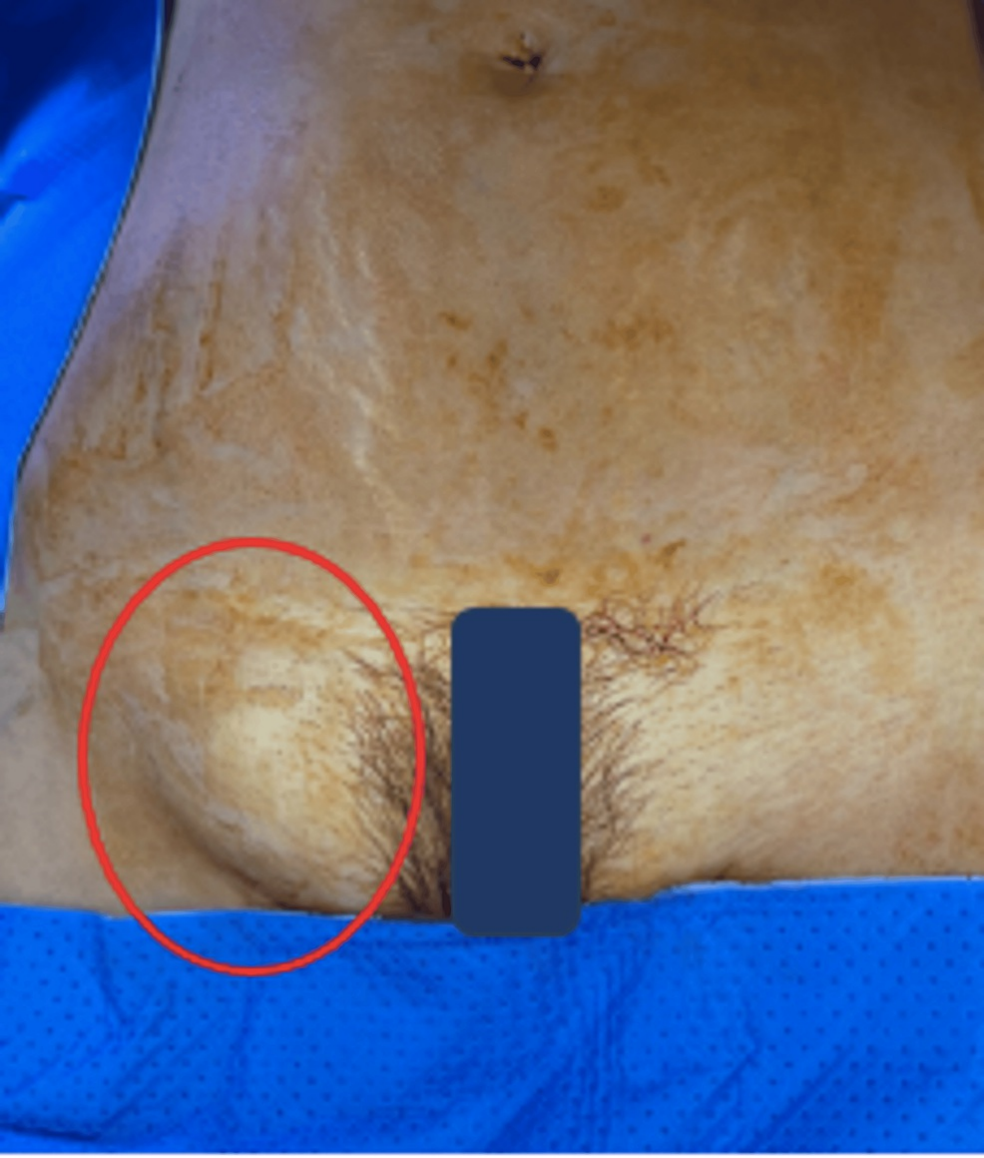
A preoperative picture of an incarcerated right femoral hernia

A 50-year-old female presented to the emergency department with a two-day history of painful right groin swelling with no associated fever, abdominal distension, vomiting, or obstipation. She is fit and well, with no significant past medical or surgical history. On examination, the patient was hemodynamically stable, with a blood pressure of 110/80 mmHg and a heart rate of 80 beats per minute. Her temperature was noted to be 36.8°C, and she was saturating at 99% on room air. Her abdominal examination showed a firm, tender, irreducible right groin mass (Figure 1). The rest of the abdominal examination was unremarkable.

She underwent a series of laboratory tests that have been detailed in Table 1. The results showed normal white cell count (WCC), neutrophils, and C-reactive protein (CRP). Her liver function tests and kidney function tests were unremarkable and within normal limits.

**Table 1:** Laboratory investigations at the time of presentation

Laboratory investigations	Results	Normal range
White cell count	8.6 x10^9^/L	4-11 x10^9^/L
Neutrophils	5.8 x 10^9^/L	2-8 x10^9^/L
Hemoglobin	121 g/L	130-180 g/L
C-reactive protein	1.6 mg/L	<5 mg/L
Bilirubin	4 umol/L	<20 umol/L
Creatinine	67 umol/L	60-110 umol/L

**Figure 2:**
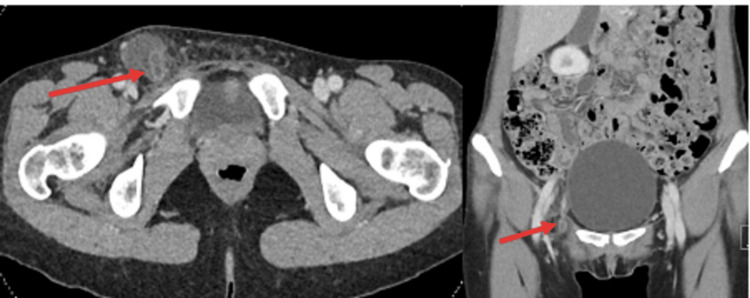
Preoperative computed tomography showed a right femoral hernia containing acute appendicitis

**Figure 3:**
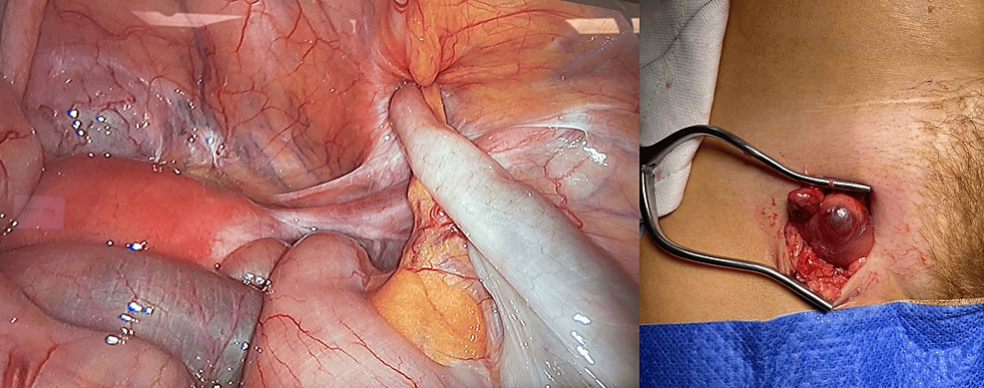
Intraoperative pictures of De Garengeot’s hernia

The patient underwent a computed tomography (CT) scan with portal phase venous contrast, which revealed the presence of a right femoral hernia containing an appendix with acute appendicitis inside the hernia (Figure 2). After obtaining informed consent, the patient underwent a laparoscopic appendicectomy and open primary repair of the femoral hernia without the use of mesh to minimize the risk of infection. The operation proceeded without any immediate intraoperative complications. During the surgery, it was observed that the appendix was grossly inflamed and trapped within the femoral hernia sac (Figure 3). 

**Figure 4:**
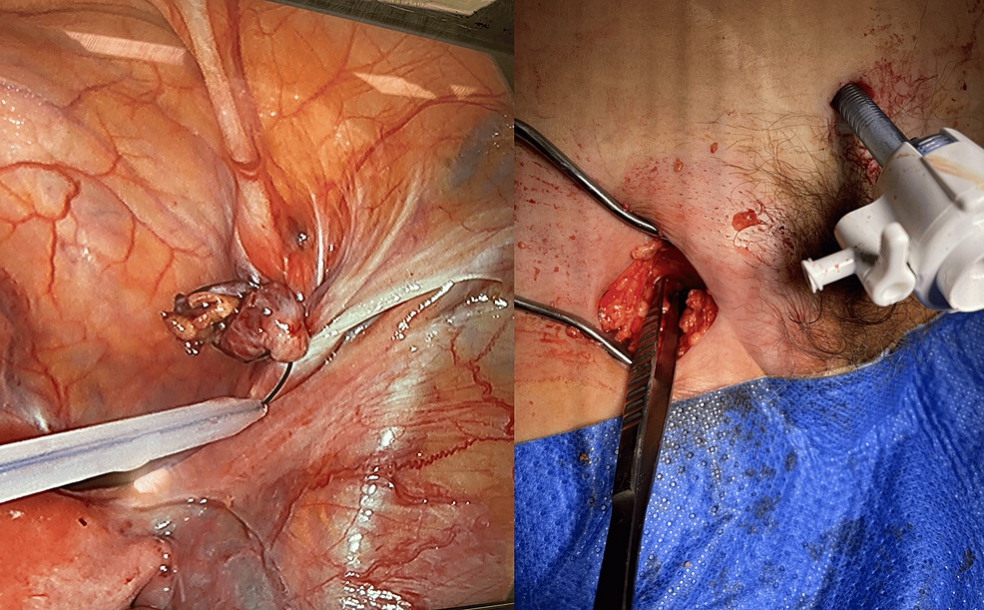
Intraoperative pictures of laparoscopic appendicectomy and exposure of the femoral hernial defect before primary repair

The appendix was carefully reduced into the abdominal cavity, excised, and retrieved through the umbilical port. Subsequently, the femoral hernia sac was dissected up to its neck and excised using a low-incision open technique. The hernial sac was then closed internally with the assistance of an endo-loop laparoscopically. Finally, the hernial defect was repaired through primary closure (Figure 4). The patient had an uneventful postoperative period.

## Discussion

De Garengeot hernia is infrequent and typically detected only during surgery for patients with incarcerated femoral hernias [6]. Femoral hernias make up approximately 4% of all groin hernias. It's important to note that the hernia sac can potentially contain various intraabdominal contents, including the omentum. Notably, a pelvic appendix carries the highest risk of entering a femoral hernia sac [7]. There is a predisposition to that type of hernia in females, with a ratio of 1:13 women affected, likely due to the increased occurrence of femoral hernias in postmenopausal women [8]. The clinical picture of acute appendicitis within the femoral hernia includes a painful, tender, and erythematous groin mass [9]. An advantageous aspect of these hernias is that, as the femoral hernia neck is tight, it prevents the extension of the infection into the peritoneal cavity. Consequently, peritonitis tends to remain localized primarily within the hernia sac [10,11].

Radiological investigations often lack specificity. While computerized tomography (CT) can aid in establishing a preoperative diagnosis and assist in surgical planning, it doesn't alter the surgical approach, which remains indicated for cases of incarcerated hernia [2,8]. In our specific case, CT proved to be highly beneficial in terms of preoperative diagnosis and facilitating surgical planning. The treatment approach involves emergency surgery. Various surgical techniques have been employed and deemed acceptable in the past, including performing an appendectomy first followed by hernia correction as a separate procedure, conducting a laparotomy for appendectomy and hernia correction, or even performing an appendectomy through the hernia sac itself while simultaneously addressing the femoral hernia during the same surgical procedure [12].

The selection of a repair method for a femoral hernia that includes a pathological appendix is a matter of debate. Typically, prosthetic material is not the preferred choice at a contaminated surgical site due to the risk of infection. However, some reports have mentioned the use of mesh repair even in cases involving an inflamed appendix, and these cases have not resulted in postoperative infections [13]. In our specific case, the appendix was laparoscopically reduced and excised, followed by the primary open repair of the hernia.

## Conclusions

In summary, De Garengeot hernia is an uncommon subtype of femoral hernia in which the appendix is located within the hernial sac. The use of computerized tomography (CT) aids in diagnosis and preoperative planning. The emergence of acute appendicitis within the femoral hernia sac requires immediate surgical intervention, and a laparoscopic appendicectomy combined with open primary hernia repair is a reasonable and less invasive approach compared to laparotomy. It is advisable to avoid the use of mesh when appendicitis is present in the hernial sac to mitigate the risk of infection.
